# Two Techniques of Tonsillectomy Performed in Identical Twins: A Case Report

**Published:** 2017-01

**Authors:** Ali Bagherihagh, Seyed Mousa Sadr Hossein

**Affiliations:** 1 *Department of Otorhinolaryngology, Baqiyatallah University of Medical Sciences, Tehran, Iran. *; 2 *Department of Otorhinolaryngology, Tehran University of Medical Sciences, Tehran, Iran.*

**Keywords:** Cautery, Dissection, Tonsillectomy, Twins

## Abstract

**Introduction:**

Cold dissection (CD) and bipolar cautery dissection (BCD) techniques are two common surgical tonsillectomy procedures used in the clinic. Obstruction has become more prevalent as the major surgical indication and is most prominently observed in younger children.

**Case Report:**

In this report, we aimed to explain the abovementioned surgical techniques in detail and compare the results in identical twins (monozygote twins). Using low energy electrocautery, irrigating the operation site continuously during cauterization, avoiding unnecessary sutures, and direct cautery to the tonsil bed are all effective measures that have diminished post-op pain in bipolar electrocautery dissection, compared to cold dissection tonsillectomy.

**Conclusion:**

Bipolar cautery dissection with some modification is very good alternative for tonsillar surgery.

## Introduction

Tonsillectomy is one of the most frequently used and the oldest surgical procedure in otorhinolaryngology (1). Various surgical techniques are usually performed during this operation, including classic blunt dissection, guillotine excision, electrocautery, cryosurgery, coblation, ultrasonic removal, laser removal, monopolar and bipolar dissection, thermal welding tonsillectomy, and ligature tonsillectomy (2). In an article published in 1986, Gates and Folbre reported recurrent tonsillitis to be the most prevalent indication for tonsillectomy (T). Chronic infection had been the primary surgical indication for T or adenotonsillectomy (AT) since the 1950s and 1980s (3). The introduction of antibiotics made infection a less common indication and consequently reduced surgical procedures (4). Indications for T or AT have changed over the past several decades. Obstruction has become more prevalent as the major surgical indication and is most prominently observed in younger children. The incidence of infections has declined, though it remains a more important indication for surgery in older children. Primary care providers and otolaryngologists must be aware of the current indications and special attention must be paid to evaluate cases of children with sleep disorder breathing to initiate appropriate management.

Cold dissection (CD) and bipolar cautery dissection (BCD) techniques are two common surgical tonsillectomy procedures used in the clinic. In the BCD technique, reduced bleeding volume is a remarkable advantage, however it coincides with post operative pain which makes it less applicable in most cases. In our experience, we the conclusion that, using modified techniques with lower thermal trauma to the tonsil bed will significantly reduce post-op pain. In this report, we aimed to explain the abovementioned surgical techniques in detail and compare the results in identical twins. 

## Case Report

Two identical twins, aged 6, with severe obstruction signs of progressive snoring and mouth breathing for two years were entered the study. According to their parents, the twins suffered from recurrent tonsillitis with five to six attacks of infectious tonsillitis during the last two years. We use the Brodsky and coworkers tonsil hypertrophy scale. In this scale, 0 indicates no tonsillar impingement on the airway; 1+ indicates less than 25% airway obstruction; 2+ indicates 25% to 50% airway obstruction; 3+ indicates 50% to 75% airway obstruction; and 4+ indicates more than 75% airway obstruction.

Physical study showed large tonsils of grade 4 according to Brodsky and coworkers scale. The twins had kissing tonsils with hyponasal speech without any obstruction in the anterior rhinoscopy. They didn’t have submucosal or overt cleft palate. Due to severe and remarkable obstructive signs, the lateral cervical x-ray was abandoned.

Blood coagulation screen was in the normal range. The twins underwent general anesthesia with the same anesthetics, same size tracheal intubations, and the same otolaryngology surgeon. Routine adenoidectomy with curette was performed and the nasopharynx was packed. 

In the first twin, routine cold dissection tonsillectomy was performed. The tonsils were grasped and medialized with a special curved Allis clamp. After anterior tonsillar pillar incision, with a décoller, tonsils were extracapsullarly dissected. Hemostasis was carried out in the bleeding sites over the tonsillar beds with 3-0, 26 G needle chromic catgut sutures. In the other twin, bipolar electrocautery tonsillectomy was performed in which the tonsils were medialized right away with an Alice clamp. Then the anterior tonsillar pillar was excised with cautery to expose the extracapsular tonsil plane. Fibrovascular bundles were delicately coagulated and dissected. Low energy bipolar cautery technique of 25 watts was used to reduce heat trauma to the tonsillar bed and consequent post-op pain. Scrub nurse normal saline continuous irrigation was also used during the surgery. Tonsillar pericapsular plane dissection was also bluntly performed. Vascular bundles attached to the tonsillar capsule and away from the bed were coagulated under a surgical loop to prevent heat trauma to the tonsillar bed. Therefore, there was no bleeding in the tonsillar bed to cauterize. In the end, after a successful hemostasis, all gauze packings were removed along with the Boyle Davis mouth gag and the patient was extubated. Total surgery time from the Boyle Davis mouth gag insertion until its removal and the operation blood loss) the suction bottle total liquid volume minus irrigation normal saline volume) were recorded. Post-op diet was started with cold liquids and vanilla ice cream around 3 to 5 hours later. Cephalexin and acetaminophen syrups were prescribed as the antibiotics and pain killers respectively and were prescribed based on the body weight. The twins were released the day after the operation. Subjective post-op pain was daily assessed using a visual analog scale based on face sensation (from crying to happiness scored 1 to 10), earache, verbal condition, and sleep quality at 1,4,7 and 10 post-op dates. Scores were recorded in the morning before pain killer use. In the first week of post-op visits, the parents were asked about the twins’ sleep quality (the number of nightly awakening pain attacks and the comfort of sleep) during the first three post-op dates and the day when regular diet started. The operation time was 20 minutes for the bipolar dissection while it was 32 minutes for the cold dissection. Moreover, intraoperative blood loss was 50 ml for the cold dissection and 5 ml for the bipolar dissection. None of the patients experienced primary or secondary hemorrhage. On the first post-op day, the twins’ mother was asked about pain and its quality based on a visual analog scale. Interestingly, the child that underwent a bipolar electrocautery tonsillectomy method got 3 while in the cold tonsillectomy method, the child got 7. According to the twins’ mother, the child that underwent the bipolar electrocautery tonsillectomy method awoke 2 hours after the operation and was able to ingest ice cream and liquids without vomiting or nausea and slept well until the next morning. However, the twin who underwent the cold dissection method was only able to start the P.O. diet 3.5 hours later due to nausea and was restless due to pain and woke up twice during the night. 

Verbal communication was dramatically reduced in this technique during the first post-op night. In the first weekly visit, pain and sleep quality of the 4th post-op date was evaluated. The pain score on the 4th and 7th post-op dates in the bipolar electrocautery technique was lower compared to the cold dissection technique (Table.1). A regular diet was started on the 5th post-op date in the bipolar electrocautery technique while in cold dissection technique; it was initiated on the 7th post-op date. Sleep quality in the bipolar electrocautery technique was also better compared to the cold dissection technique. Interestingly, sleep and pain quality in the 2nd post-op week was similar in the twins.

**Table1:** Post operative pain score (VAS)

**Method**	**VAS 1st**	**VAS 4** ^th^	**VAS 7th**	**VAS 14th**
Bipolar	3	3	2	1
Cold dissection	7	6	4	1

VAS=visual analogue score

## Discussion

Tonsillectomy is one of the most common surgeries worldwide (1). Cold dissection and electrocautery dissection are the main and most commonly used techniques for tonsillectomy (2). However, the cold dissection technique is usually performed for residents and for educational aims. Microdebrider, coblation, radiofrequency, laser and monopolar electrocautery are other surgical techniques used for tonsillectomy. Post-op pain is one of the most vexing tonsillectomy side effects and is reportedly the most common cause for patients’ referral during the first two weeks of post-op visits (5). Pain is usually caused by heat or mechanical trauma to adjacent tissues that is eventually accompanied by edema, pharyngeal muscles spasm, and tonsillar bed nerve endings irritation (6). Regarding the annoying nature of post tonsillectomy pain, many studies have been developed to compare the pain severity of different surgical techniques. Although bipolar electrocautery dissection has less intraoperative bleeding, controversial findings have been reported regarding its post-op pain severity and patient comfort. Leinbach et al, in their systematic review, reported more severe post-op pain in the electrocautery dissection technique tonsillectomy than in the cold dissection tonsillectomy technique (7). In Mahmut Ozkiris study, the post-op pain score was also reported to be higher in the electrocautery dissection technique than in the cold dissection and thermal welding tonsillectomy techniques (8). Conversely, Raut et al showed that the post-op pain score in the electrocautery dissection tonsillectomy technique was not statistically different with cold dissection. They claimed that the electrocautery dissection was an ideal method for pediatric tonsillectomy due to lessened bleeding and a shorter operation time (9). Silveira H et al reported signigicantly more post-op pain in the electrocautery dissection technique tonsillectomy compared to the cold dissection tonsillectomy technique. They also suggested that electrocautery dissection tonsillectomy was a suitable technique in very low age pediatric patients due to their low blood volume (1). Kereschner et al reported that most comparative studies on electrocautery and cold dissection techniques either lacked precise explanations of surgical details or used high energy cautery. They also added that holistic studies comparing low energy electrocautery and cold dissection tonsillectomy techniques were brief (10). This issue has especially been cleared where lower post op-pain and faster recovery has been reported in one study with low energy electrocautery tonsillectomy (11). In another randomized study in which coblation tonsillectomy was compared with low energy electrocautery tonsillectomy, no significant difference was reported regarding post-op pain and the ability to start a regular diet (12). The advantage of the present study is the use of monozygotic twins. Their identical appearance and psychological characteristics handed us a unique opportunity to precisely compare post-op pain and quality of life between two common techniques of tonsillectomy. In our previous experiences, we have taken some measures to reduce heat trauma to the bed of the tonsils and noticed low pain and less inconvenience during the post operative period of the bipolar electrocautery dissection tonsillectomy. Therefore, we wished to test it on identical twins by comparing it with the cold dissection tonsillectomy technique. Our findings showed that the cold dissection tonsillectomy technique, especially when a lot of surgical sutures are performed in order to acquire better hemostasis, causes remarkable pain. As mentioned earlier, low energy electrocautery, continuous saline irrigation of the operation site during cauterization, and avoiding unnecessary sutures and direct cautery to the tonsillar bed are all effective measures that have diminished post-op pain in bipolar electrocautery dissection compared to cold dissection tonsillectomy techniques.

## Conclusion

Our study compared two different surgical tonsillectomy techniques on identical twins and showed that the intensity of post-op pain was drastically reduced when using appropriate measures. It seems that the bipolar electrocautery dissection technique is an appropriate tonsillectomy method because of its low intrao- perative bleeding and reduced operation time.
